# Accurate Genomic Predictions for Chronic Wasting Disease in U.S. White-Tailed Deer

**DOI:** 10.1534/g3.119.401002

**Published:** 2020-03-02

**Authors:** Christopher M. Seabury, David L. Oldeschulte, Eric K. Bhattarai, Dhruti Legare, Pamela J. Ferro, Richard P. Metz, Charles D. Johnson, Mitchell A. Lockwood, Tracy A. Nichols

**Affiliations:** * Department of Veterinary Pathobiology, Texas A&M University, College Station, Texas,; † Texas A&M Veterinary Medical Diagnostic Laboratory, College Station, Texas,; ‡ Genomics Core, Texas A&M AgriLife Research, College Station, Texas,; § Texas Parks and Wildlife Department, Austin, Texas, and; ** USDA-APHIS-VS-Cervid Health Program, Fort Collins, CO

**Keywords:** genome-wide association, chronic wasting disease, white-tailed deer, genomic prediction, heritability

## Abstract

The geographic expansion of chronic wasting disease (CWD) in U.S. white-tailed deer (*Odocoileus virginianus*) has been largely unabated by best management practices, diagnostic surveillance, and depopulation of positive herds. Using a custom Affymetrix Axiom single nucleotide polymorphism (SNP) array, we demonstrate that both differential susceptibility to CWD, and natural variation in disease progression, are moderately to highly heritable ($h^{2}=0.337\pm 0.079─0.637\pm 0.070)$ among farmed U.S. white-tailed deer, and that loci other than *PRNP* are involved. Genome-wide association analyses using 123,987 quality filtered SNPs for a geographically diverse cohort of 807 farmed U.S. white-tailed deer (n = 284 CWD positive; n = 523 CWD non-detect) confirmed the prion gene (*PRNP*; G96S) as a large-effect risk locus (*P*-value < 6.3E-11), as evidenced by the estimated proportion of phenotypic variance explained (PVE ≥ 0.05), but also demonstrated that more phenotypic variance was collectively explained by loci other than *PRNP*. Genomic best linear unbiased prediction (GBLUP; n = 123,987 SNPs) with *k*-fold cross validation (*k* = 3; *k* = 5) and random sampling (n = 50 iterations) for the same cohort of 807 farmed U.S. white-tailed deer produced mean genomic prediction accuracies ≥ 0.81; thereby providing the necessary foundation for exploring a genomically-estimated CWD eradication program.

The fatal wasting syndrome known as chronic wasting disease (CWD) was first observed among captive mule deer (*Odocoileus hemionus*) and black-tailed deer *(Odocoileus hemionus columbianus*) within several Colorado wildlife facilities during the late 1960s, and histologically recognized as a prion disease by the late 1970s (Williams and Young 1980; Moreno and Telling 2018). Thereafter, CWD was described in free-ranging U.S. mule deer, elk (*Cervus elaphus nelsoni*), white-tailed deer (*Odocoileus virginianus*; hereafter WTD) and moose (*Alces alces shirasi*), with subsequent diagnostic surveillance suggesting an irreversible geographic expansion of the disease among farmed and free-ranging populations of these species (Moreno and Telling 2018; Gavin *et al.* 2019; Osterholm *et al.* 2019). At present, at least 26 U.S. states and multiple Canadian provinces are known to be affected by CWD (Moreno and Telling 2018; Gavin *et al.* 2019; Osterholm *et al.* 2019). Likewise, Norway, Finland, and the Republic of Korea have also reported CWD in free-ranging reindeer (*Rangifer tarandus*; Norway), moose (Norway, Finland), and imported elk (Korea) (Moreno and Telling 2018; Gavin *et al.* 2019; Osterholm *et al.* 2019). The implementation of modern best management practices, including containment and depopulation of positive herds, has not prevented the emergence of CWD in new geographic areas (Moreno and Telling 2018; Gavin *et al.* 2019; Osterholm *et al.* 2019). Therefore, a need currently exists to develop novel strategies to reduce the prevalence of CWD among farmed deer and elk.

## Materials And Methods

### Study overview

Herein, we investigate differential susceptibility to CWD among farmed U.S. WTD by utilizing genomic DNA samples from CWD positive and CWD non-detect WTD to perform next-generation sequencing with variant prediction for the construction and validation of a medium density SNP array. Thereafter, we use the array in conjunction with *PRNP* genotypes to conduct genome-wide association analyses (GWAA’s) and produce marker-based heritability estimates. Finally, we conclude our study by utilizing the genome-wide SNP data to deploy genomic prediction equations with *k*-fold cross validation to assess the potential for developing a genomically-estimated CWD eradication program.

### Animal resources, CWD diagnostics, and DNA isolation

Frozen whole blood samples (n = 448) and rectal biopsies (n = 37) from farmed U.S. white-tailed deer (WTD; both sexes) were available within an existing USDA APHIS repository that was created via federal CWD surveillance activities; including depopulations of CWD positive herds (USDA APHIS, Fort Collins, CO). All herds included both CWD positive (n = 256) and CWD non-detect (n = 229) WTD (Thomsen *et al.* 2012), with geographic representation that included WTD farms located in the U.S. Midwest, Northeast, and South. All diagnostic classifications were based upon immunohistochemistry (*i.e.*, IHC of lymph node, obex), as implemented and performed at USDA National Veterinary Services Laboratory (NVSL) in Ames Iowa (Thomsen *et al.* 2012). Genomic DNA was isolated from frozen whole blood using the Applied Biosystems MagMAX DNA Multi-Sample Ultra Kit with the KingFisher 96 Purification System (ThermoFisher), as recommended by the manufacturer, at the Texas Veterinary Medical Diagnostic Laboratory (TVMDL; College Station, TX). Genomic DNA was isolated from WTD rectal biopsies using the LGC sbeadex tissue purification kit (LGC) with automation at GeneSeek Neogen (Lincoln, NE). Hair samples (n > 700) from farmed WTD (both sexes) were also available within an existing Texas Parks and Wildlife Department (TPWD; Austin, TX) repository created via surveillance and depopulation efforts after the initial detection of CWD in Texas. At the time of study, sample repositories for these herds included CWD positive (n ≥ 100) and CWD non-detect (n ≥ 600) WTD, with a geographic representation that included WTD farms located in the U.S. South (Texas). All diagnostic classifications for TPWD samples were based upon IHC (*i.e.*, lymph node, obex, and one tonsil) initially performed at TVMDL, with further confirmation at NVSL (Thomsen *et al.* 2012). Genomic DNA was isolated from WTD hair follicles using the LGC sbeadex tissue purification kit (LGC) with automation at GeneSeek Neogen (Lincoln, NE). All genomic DNAs were quantified and evaluated for purity (260/280 ratio) via Nanodrop (ThermoFisher).

### Reduced representation libraries and sequencing

Pooled DNA samples were previously shown to be effective for variant prediction; thus enabling downstream genotyping in WTD (Seabury *et al.* 2011). Therefore, pooled DNA samples were created for CWD positive (WT1) and CWD non-detect (WT0) WTD acquired from the USDA APHIS repository (frozen blood). Briefly, WT1 (n = 190) and WT0 (n = 184) genomic DNAs with concentrations ≥ 15 ng / µl were used to construct sequencing pools representing depopulated WTD from the U.S. Midwest, Northeast, and South by targeting 50 ng of genomic DNA per WTD in each respective pool (WT1, WT0). Genomic DNAs with concentrations < 15 ng / µl were retained for downstream Affymetrix Axiom and *PRNP* genotyping. Aliquots from each genomic DNA pool (WT0, WT1) were digested with EcoRI, HindIII, and PstI (NEB) in 1X CutSmart buffer for 4 hr at 37°. Enzymes were heat inactivated at 80° for 20 min and held at 10° until ligation. Ligase buffer, ligase (NEB) and barcoded enzyme-specific adapters compatible with DNA possessing EcoRI, HindIII or PstI overhangs were added to the digested samples, and incubated at 16° for 8 hr. Following heat inactivation at 80° for 20 min, 1/10^th^ volume of 3M NaAc (pH 5.2) and two volumes of 100% ethanol were added to each sample, and then held at -20° for 1 hr before spinning at high speed for 10 min in a bench-top microfuge. Pellets were washed twice in 1 ml 70% ethanol and resuspended in 130 µl 1X TE. Samples were then sheared to an average size of 350 bp on the Covaris E220 sonicator, and AMPure XP bead purified as per the manufacturers protocol (Beckman Coulter). Sheared DNA fragments were size selected using a Pippin Prep 2% dye-free agarose gel with internal size markers (Sage Bioscience); aiming for 300-800 bp inserts. Recovered samples were cleaned with 1X AMPure XP beads and end-repaired first with Bst DNA Polymerase (NEB), then with a DNA End Repair Kit (NEB), and A-Tailed using Klenow Fragment (3′→5′ exo-) (NEB) in the presence of 50 nM ATP. An Illumina P7-adapter (Adapter B) was ligated to the A-tailed ends as described above. Following two rounds of AMPure XP bead purification, 150 ng of each pool was then subjected to “pre-selection PCR” (PreCR) in which a biotinylated forward primer (P5-Select) and unique indexed reverse primers (TDX) were used to amplify and tag desired DNA fragments. Reactions (200µl total) contained 200 nM dNTPs, biotinylated forward and two P7-index primers per pool, 4 units Q5 Hi-Fidelity Taq (NEB), and were split into 2 X 100 µl volumes for thermocycling. Following an initial denaturation at 98° for 30 sec, samples were subjected to 15 cycles of 98° for 10 sec, 72° for 30 sec then a final elongation at 72° for 5 min and held at 4°. PCR products were purified using Qiagen PCR purification columns, then 1X AMPure XP beads, and quantified via DeNovix. Removal of undesirable fragments (P5 to P5 and P7 to P7 ligated products) was achieved with Dynabeads M-270 Streptavidin coupled magnetic beads (ThermoFisher). Briefly, 50 µl of beads per sample were captured and washed twice with 1X Bead Washing Buffer (1X BWB, 10 mM Tris-HCl with pH 7.5, 1 mM EDTA, 2 M NaCl). Beads were resuspended in 100µl 2X BWB and mixed with 2000 ng of PreCR product in 100 µl EB. After 20 min at room temperature, beads were captured and washed three times in 200 µl 1X BWB, twice in 200 µl water, and once in 100 µl 1X SSC. Beads were then resuspended in 50 µl 1X SSC, heated at 98° for 5 min, and placed on a magnet, with the supernatant removed thereafter. This elution was repeated and the final supernatants were purified with Qiagen PCR columns, as recommended by the manufacturer. The eluted ssDNA was DeNovix quantified, and diluted to 1 ng/µl with EB. A final PCR was performed on 10 ng of input DNA using FiLi-F1 and FiLi-R1 primers in a 50 µl reaction, with only 8 cycles. Final PCR products representing WT0-EcoRI, WT0-HindIII, WT0-PstI, WT1-EcoRI, WT1-HindIII, and WT1-PstI were purified with 1X AMPure XP beads, quantified and assessed for quality on a Fragment Analyzer (Advanced Analytics), and sequenced (2 × 125 bp, paired end) on the Illumina HiSeq 2500 at the Texas AgriLife Genomics and Bioinformatics Core Facility at Texas A&M University. Raw reads generated for each library were as follows: WT1 EcoRI (134,299,714); WT1 PstI (175,412,740); WT1 HindIII (152,371,052); WT0 EcoRI (145,989,752); WT0 PstI (120,598,450); WT0 HindIII (137,148,058). Primers used were as follows: For ligation to restriction enzyme cut DNA, adapters were made by mixing equimolar amounts of top (T) and bottom (B) oligos in 1X oligo hybridization buffer (50 mM NaCl, 1 mM EDTA, 10 mM Tris-HCl, pH 8.0), heating them to 98° for 1 min, and allowing them to cool to room temperature at a rate of 0.1° per second. Primer sequences used were as follows (X denotes bases used for barcoding): Eco_T,5′-AAT GAT ACG GCG ACC ACC GAG ATC TAC ACX XXX XXX XAC ACT CTT TCC CTA CAC GAC GCT CTT CCG ATC T-3′; Eco_B,5′-AAT TAG ATC GGA AGA GCG TCG TGT AGG GAA AGA GTG TXX XXX XXX GTG TAG ATC TCG GTG GTC GCC GTA TCA TT-3′; Hind_T, 5′-AAT GAT ACG GCG ACC ACC GAG ATC TAC ACX XXX XXX XAC ACT CTT TCC CTA CAC GAC GCT CTT CCG ATC T-3′; Hind_B, 5′-AGC TAG ATC GGA AGA GCG TCG TGT AGG GAA AGA GTG TXX XXX XXX GTG TAG ATC TCG GTG GTC GCC GTA TCA TT-3′; Pst_T, 5′-AAT GAT ACG GCG ACC ACC GAG ATC TAC ACX XXX XXX XAC ACT CTT TCC CTA CAC GAC GCT CTT CCG ATC TTG CA-3′; Pst_B, 5′- AGA TCG GAA GAG CGT CGT GTA GGG AAA GAG TGT XXX XXX XXG TGT AGA TCT CGG TGG TCG CCG TAT CAT T-3′; Adaptor-B_T, /5Phos/GAT CGG AAG AGC ACA CGT CTG AAC TCC AGT CAC-3′; Adaptor-B_B, 5′-GTG ACT GGA GTT CAG ACG TGT GCT CTT CCG ATC T-3′; P5_Select, /5BiotinTEG/AAT GAT ACG GCG ACC ACC GAG ATC TAC AC-3′; FiLi-F1: 5′-AAT GAT ACG GCG ACC ACC GAG ATC TAC AC-3′; FILi-R1: 5′-CAA GCA GAA GAC GGC ATA CGA GAT-3′; TDX, 5′-CAA GCA GAA GAC GGC ATA CGA GAT XXX XXX XGT GAC TGG AGT TCA-3′.

### Sequence analysis and affymetrix axiom array design

All Illumina sequences were trimmed for quality and adapters using CLC Genomics Workbench 10.1.1 (Qiagen), as previously described (Halley *et al.* 2014; Halley *et al.* 2015; Sollars *et al.* 2017). All trimmed reads were mapped to the WTD genome assembly (GCF_002102435.1 Ovir.te_1.0; https://www.ncbi.nlm.nih.gov/assembly/GCF_002102435.1/) using the CLC Genomics Workbench 10.1.1 reference mapping algorithm (Seabury *et al.* 2011; Halley *et al.* 2014; Halley *et al.* 2015; Sollars *et al.* 2017). Variant prediction was performed using a probabilistic approach implemented within CLC Genomics Workbench 10.1.1 (Halley *et al.* 2014; Halley *et al.* 2015; Oldeschulte *et al.* 2017; Sollars *et al.* 2017). This algorithm estimates error probabilities from quality scores, and uses these probabilities to determine the most likely allele combination per nucleotide position, thus facilitating a user-specified minimum probability threshold (*P* ≥ 0.95) for variant prediction, and variant quality scores (Halley *et al.* 2014; Halley *et al.* 2015; Oldeschulte *et al.* 2017; Sollars *et al.* 2017). Additional variant prediction parameters and filters were similar to those recently described (Seabury *et al.* 2011; Oldeschulte *et al.* 2017), and the probabilistic approach produced evidence for 6,268,706 variants, which included 5,561,550 putative SNPs (*P* ≥ 0.95; Minor Allele Frequency ≥ 0.01). Variant prediction results were exported from CLC as a single variant call formatted file (VCF), which was used for SNP array design. Briefly, the VCF file was filtered according to the Affymetrix Axiom myDesign guidelines for SNP submission (http://www.affymetrix.com/support/technical/byproduct.affx?product=axiom_custom_agrigenomics) using a custom python script as follows: Retain only biallelic SNPs with minimum depth = 10, maximum depth = 150, minimum minor allele frequency (MAF) ≥ 0.15, minimum SNP quality score = 30, identify probe overlaps for exclusion, and prioritize variants that maximize array density (*i.e.*, A/T and C/G take up two spaces on the array). The python script as well as more detailed documentation are available in Additional File 30 (DRYAD). The targeted number of SNPs for submission to Affymetrix was > 200,000; to facilitate internal Affymetrix scoring (*i.e.*, by pconvert, best_pconvert; recommended, neutral, or not recommended) that would enable the design of a final Affymetrix Axiom 200K SNP array. Collectively, 200,000 SNPs were favorably scored (n = 179,508 recommended; n = 20, 492 neutral) and used for array fabrication.

### *PRNP* and affymetrix axiom array genotyping

*PRNP* genotyping for missense variants at codons 37, 95, 96, 116, and 226 was performed at GeneSeek Neogen (Lincoln, NE) as part of an existing commercial genotyping by sequencing (GBS) service. Briefly, the functional *PRNP* gene was PCR amplified using primers designed to be exclusionary to a processed pseudogene as previously described (O’Rourke *et al.* 2004), and the resulting amplicons were purified via AMPure XP beads as recommended by the manufacturer (Beckman Coulter); thus allowing for the creation of barcoded Illumina Nextera XT DNA libraries and amplicon sequencing on an Illumina MiSeq. *PRNP* genotypes were called from the aligned read pileups at GeneSeek Neogen, and delivered in text format. Affymetrix Axiom 200K genotyping was also performed at GeneSeek Neogen using the established Affymetrix best practices workflow; with genotypes delivered in text format. Affymetrix quality control thresholds were DQC ≥ 0.82, QC call rate ≥ 97%, passing samples in the project ≥ 95%, and average call rate for passing samples ≥ 97%. Collectively, 860 WTD samples with the desired metadata (*i.e.*, sex, age, U.S. general region) passed all Affymetrix QC filters; each with 125,968 SNP array genotypes, and paired *PRNP* genotypes, thus yielding a combined set of 125,973 SNP genotypes for analysis. Fifty-three WTD did not have CWD diagnostic data at the time of study. SNPs which did not convert on the Affymetrix Axiom 200K WTD array were primarily due to call rates below the best practices threshold (n = 37,197), and failures to meet thresholds in two or more best practices criteria (n = 36,045). All SNP conversion types are comprehensively summarized in DRYAD (Additional File 32).

### GWAA and genomic prediction with cross validation

Prior to all analyses, a comparative marker map was created by aligning the WTD *PRNP* sequence and all Affymetrix Axiom 200K probe sequences with ARS-UCD1.2 (GCA_002263795.2) via blastn, thus providing comparative evidence for the origin of the array SNPs (*i.e.*, autosomal *vs.* non-autosomal); which was necessary because the draft *de novo* WTD genome assembly (GCF_002102435.1 Ovir.te_1.0) is unanchored (*i.e.*, by maps or *in situ* hybridization). The comparative marker map was joined to the combined set of all genotypes (*PRNP* + Affymetrix Axiom array), and quality control analyses were performed in SVS v8.8.2 or v8.8.3 (Golden Helix). Initially, pairwise IBS distances were computed to identify twins and duplicate samples. Eight samples present in both repositories (USDA APHIS, TPWD) were purposely duplicated for use as process controls, and correctly identified by IBS/IBD estimates (Additional File 31 in DRYAD). Eight additional samples were also identified as either duplicates or potential twins. In all cases, only the sample with the highest call rate was retained for further analyses. Additional quality control analyses and filtering were as follows: sample call rate (< 97% excluded), and thereafter, SNP filtering by call rate (> 15% missing excluded), MAF (< 0.01 excluded), polymorphism (monomorphic SNPs excluded), and Hardy-Weinberg Equilibrium (excludes SNPs with HWE *P*-value < 1e-25), which yielded 123,987 SNPs for all analyses. *PRNP* SNPs which failed to endure quality control filtering included codons 95 (MAF < 0.01) and 116 (monomorphic), whereas codons 37, 96, and 226 remained. All GWAA’s and genomic predictions with *k*-fold cross validation were performed on 807 WTD with recorded metadata that included sex, age, U.S. general region of origin (*i.e.*, Midwest, Northeast, South), and CWD diagnostics. CWD phenotypes used in all analyses were: CWD Scores (0 = non-detect, 1 = lymph node positive, 2 = lymph node and obex positive); CWD Binary (0 = non-detect, 1 = lymph node positive and/or obex positive). However, at the time of study, one WTD (sample ID: MQ6Q) only possessed diagnostic data for a CWD positive tonsil biopsy, and thus was assigned a CWD Score and a CWD Binary phenotype of “1”. All WTD GWAA’s were performed using a mixed linear model with variance component estimates, as described and implemented in EMMAX, and executed in SVS v8.8.2 or v8.8.3 (Golden Helix), where all genotypes are also recoded as 0, 1, or 2, based on the incidence of the minor allele (Kang *et al.* 2010; Segura *et al.* 2012; Seabury *et al.* 2017; Smith *et al.* 2019). Briefly, the general mixed model can be specified as: y=Xβ+Zu+ϵ, where *y* represents a n×1 vector of observed CWD phenotypes, *X* is a n×f matrix of fixed effects, β is a f×1 vector representing the coefficients of the fixed effects, *u* represents the unknown random effect, and *Z* is a n×t matrix relating the random effect to the CWD phenotypes of interest (Kang *et al.* 2010; Segura *et al.* 2012; Seabury *et al.* 2017; Smith *et al.* 2019). Herein, it is necessary to assume that $Var\left(u\right)=\sigma_{g}^{2}K$ and $Var\left(\epsilon \right)=\sigma_{e}^{2}I$, whereby $Var\left(y\right)=\sigma_{g}^{2}ZKZ^{’}+\sigma_{e}^{2}I$, but in this study *Z* represents the identity matrix *I*, and *K* represents a relationship matrix of all WTD samples. To solve the mixed model equation using a generalized least squares approach, we must first estimate the relevant variance components (*i.e.*, $\sigma_{g}^{2}$ and $\sigma_{e}^{2}$) as previously described (Kang *et al.* 2010; Segura *et al.* 2012; Seabury *et al.* 2017; Smith *et al.* 2019). Variance components were estimated using the REML-based (restricted maximum likelihood) EMMA approach (Kang *et al.* 2010), with stratification accounted for and controlled using a genomic relationship matrix (*G*) (VanRaden 2008), as computed from the WTD genotypes. Genomic relationship matrix (GRM) heritability estimates ($\left[h^{2}={\text{σ}}_{g}^{2}/({\text{σ}}_{g}^{2}+{\text{σ}}_{e}^{2})\right]$) were produced as previously described (Kang *et al.* 2010; Segura *et al.* 2012; Seabury *et al.* 2017; Smith *et al.* 2019). Moreover, because previous WTD studies indicate that the probability of CWD infection increases with age (Grear *et al.* 2006), and that CWD may disparately affect male and female WTD in different U.S regions, including differences in clinical disease progression and mortality (Grear *et al.* 2006; Edmunds *et al.* 2016), the possibility for different CWD strains must be considered (Bistaffa *et al.* 2019). Therefore, the following fixed-effect covariates were specified for comparison of GWAAs: sex, age, U.S. region of origin, and the total number as well as types (0 = none-detected; 1 = lymph node only; 2 = lymph node and obex) of CWD positive diagnostic tissues, with one exception (*i.e.*, sample ID: MQ6Q), as noted above.

For all genomic prediction analyses involving *k*-fold cross validation, we used GBLUP as previously described (Taylor 2014) and implemented in SVS v8.8.2 or v8.8.3 (Golden Helix), where the variance components were again estimated using the REML-based EMMA technique (Kang *et al.* 2010) with a genomic relationship matrix (*G*) (VanRaden 2008; Taylor 2014). For WTD GBLUP analyses, consider the general mixed model equation: $y=X_{f}B_{f}+u+\epsilon$, across *n* WTD samples where fixed effects specified as $B_{f}$ include the intercept and any additional covariates (*i.e.*, U.S. region, sex, age); but also assume $Var\left(\epsilon \right)=\sigma_{e}^{2}I$, as above, and that the random effects *u* are additive genetic merits (*i.e.*, genomically estimated breeding values or GEBVs) for these WTD samples, which are produced from *m* markers as u=Mα, where *M* is a n×m matrix, and *α* is a vector where $\alpha_{k}$ is the allele substitution effect (ASE) for marker *k*. In this study, we used overall normalization for matrix *M*, as implemented in SVS v8.8.2 or v8.8.3 (Golden Helix), and explored solutions with and without gender corrections (*i.e.*, full dosage compensation, no dosage compensation) (Taylor 2014), to produce GEBVs for all WTD samples as well as estimates of ASE for all SNPs. Moreover, considering that all training set samples precede the validation set, we define Z=[I|0], where the width and height of *I* is given as $n_{t}$, the width of the zero matrix is given as $n_{v}$, and the height of the zero matrix is $n_{t}$. Thus we can partition *u*, $X_{f}$, and *y* according to their origin (*i.e.*, training *vs.* validation set) as $u=\left[\begin{array}{c} u_{t}\\ u_{v} \end{array}\right]$, $X_{f}=\left[\begin{array}{c} X_{ft}\\ X_{fv} \end{array}\right]$, $y=\left[\begin{array}{c} y_{t}\\ y_{v} \end{array}\right]$, and compute a genomic relationship matrix using all samples for use with the EMMA technique (Kang *et al.* 2010); to implement a mixed model for the training set as follows: $y_{t}=X_{ft}B_{f}+Zu+\epsilon_{t}$, where $Var\left(u\right)=\sigma_{G}^{2}G$ and $Var\left(Zu\right)=\sigma_{G}^{2}ZGZ^{’}$. Finally, we predict the validation set phenotypes as: ${\hat{y}}_{v}=X_{fv}{\hat{B}}_{f}+{\hat{u}}_{v}$, from the intercept and any validation covariates $X_{fv}$ as well as the predicted values of ${\hat{u}}_{v}$. Additional formulae and supporting documentation are available at https://doc.goldenhelix.com/SVS/latest/svsmanual/mixedModelMethods/overview.html#gblupproblemstmt. Notably, prior to this study, GEBVs were not estimated or utilized in WTD, and thus they cannot be expected to be intuitive or easily understood by U.S. WTD farmers or relevant regulatory agencies. However, the predicted CWD binary phenotypes are both intuitive and easily understood as estimates of enhanced or reduced susceptibility to CWD. Because GBLUP predicts CWD binary phenotypes across a range of values (*i.e.*, 0 to 1), SVS v8.8.2 and v8.8.3 (Golden Helix) considers predicted values of 0.5 and higher as “1”, and < 0.5 as “0”, thus facilitating the calculation of important summary statistics which require binary classifications. Justification for rounding is evident within the histograms representing the frequency distributions of the predicted CWD binary phenotypes (Fig. S1), the relevant GEBVs (Fig. S2), and the relationship between the predicted CWD binary phenotypes and the relevant GEBVs (Fig. S3); with an obvious break that occurs at 0.50 (Fig. S1-S3). Binary summary statistics for GBLUP-based genomic predictions with *k*-fold (*k* = 3; *k* = 5) cross validations (n = 50 iterations) were computed in SVS v8.8.2 or v8.8.3 (Golden Helix) as follows: Area Under the Curve as $AUC=\frac{U_{1}}{n_{1}n_{2}}$, where $n_{1}$ is the sample size of observations with CWD binary phenotypes of 0, $n_{2}$ is the sample size of observations with CWD binary phenotypes of 1, and $U_{1}=R_{1}-\frac{n_{1}(n_{1}+1)}{2}$, where $R_{1}$ is the sum of the ranks for the predicted binary CWD phenotypes with actual phenotypes of 0 (from CWD diagnostics); Mathews Correlation Coefficient as $MCC=\frac{TP\cdot TN-FP\cdot FN}{\sqrt{\left(TP+FN\right)\cdot \left(FP+TN\right)\cdot \left(TP+FP\right)\cdot \left(FN+TN\right)}}$, where TP is the number of true CWD positives (from CWD diagnostics), TN is the number of true CWD non-detects (from CWD diagnostics), FP is the number of false positives, and FN is the number of false non-detects among the predicted CWD binary phenotypes; Genomic Prediction Accuracy as $ACC=\frac{TP+TN}{TP+FN+FP+TN}$; Sensitivity (true positive rate) as $TPR=\frac{TP}{TP+FN}$; Specificity (true negative rate) as $SPC=\frac{TN}{FP+TN}$; Root Mean Square Error as $RMSE=\sqrt{\frac{\sum_{i=1}^{n}{\left(y_{i}-{\hat{y}}_{i}\right)}^{2}}{n}}$. For the GBLUP predicted CWD Scores (0, 1, 2), we also produced and report relevant summary statistics from the *k*-fold (*k* = 3; *k* = 5) cross validations (n = 50 iterations) computed in SVS v8.8.2 or v8.8.3 (Golden Helix) as follows: Pearson’s Product-Moment Correlation Coefficient as $r_{y,\hat{y}}=\frac{\sum_{i=1}^{n}(y_{i}-\bar{y})(\hat{y_{i}}-\hat{\bar{y}})}{(n-1)s_{y}s_{\hat{y}}}$ where $s_{y}$ and $s_{\hat{y}}$ are the standard deviations; Residual Sum of Squares as $RSS=\sum_{i=1}^{n}{\left(y_{i}-\hat{y}\right)}^{2}$; Total Sum of Squares $TSS=\sum_{i=1}^{n}{\left(y_{i}-\bar{y}\right)}^{2}$; R-Squared as $R^{2}=1-\frac{RSS}{TSS}$; Root Mean Square Error as $RMSE=\sqrt{\frac{RSS}{n}}$; Mean Absolute Error as $MAE=\frac{1}{n}\sum_{i=1}^{n}\left|y_{i}-{\hat{y}}_{i}\right|$.

### Randomizing and blinding

All GBLUP-based *k*-fold (*k* = 3; *k* = 5) cross validations (*i.e.*, CWD binary; CWD-scores) were performed using automated random sampling to define the validation set (*i.e.*, to predict on) and the training set, for the specified values of *k*.

### Data availability

Accession codes are as follows: Illumina sequence data (SRA: SRR10313416-SRR10313421); genotype and phenotype data (DRYAD https://doi.org/10.5061/dryad.xd2547dcw).

## Results And Discussion

**Figure 1:**
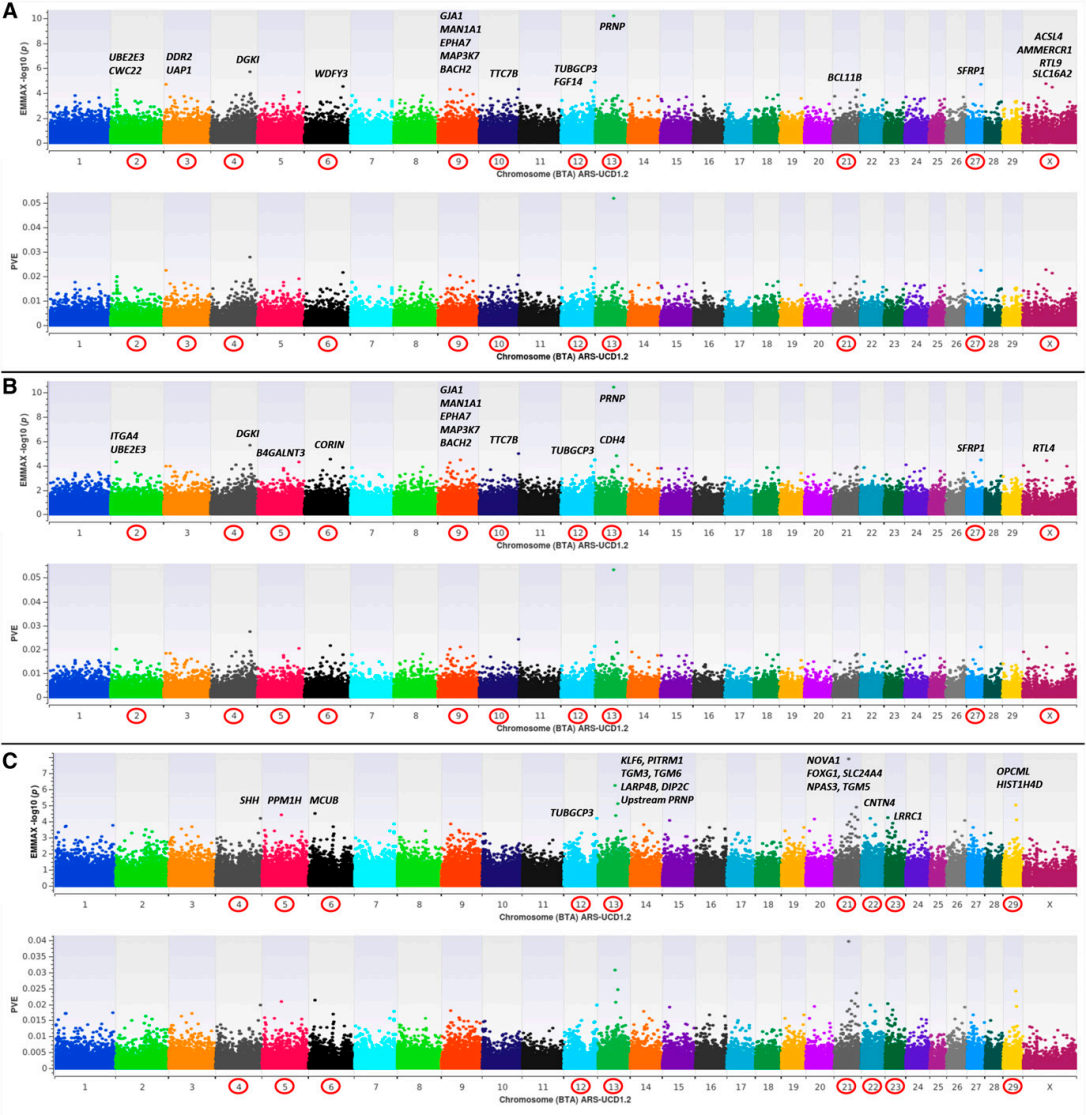
EMMAX binary case-control (0, 1) genome-wide association analyses (GWAA) for Chronic Wasting Disease (CWD) in farmed U.S. white-tailed deer (*Odocoileus virginianus*; hereafter WTD). All dual-panel manhattan plots depict -log10 *P*-values and the proportion of phenotypic variance explained (PVE) by white-tailed deer marker-effects on the y-axis, and the comparative position of all probe sequences on the x-axis, as inferred by blastn alignment with the bovine genome (ARS-UCD1.2). All analyses include diagnostically confirmed CWD positive (n = 284) and CWD non-detect (n = 523) WTD, and marker-based GRM heritability estimates ($\left[h^{2}={\text{σ}}_{g}^{2}/({\text{σ}}_{g}^{2}+{\text{σ}}_{e}^{2})\right]$) (Kang *et al.* 2010; Segura *et al.* 2012; Seabury *et al.* 2017; Smith *et al.* 2019). **a**, EMMAX GWAA for CWD with no fixed-effect covariates, high GRM heritability estimates ($h^{2}=0.637\pm 0.070$) and relevant positional candidate genes. **b**, EMMAX GWAA for CWD with fixed-effect covariates (sex, age, U.S. region), high GRM heritability estimates ($h^{2}=0.546\pm 0.076)$ and relevant positional candidate genes. **c**, EMMAX GWAA for CWD with fixed-effect covariates (sex, age, U.S. region, CWD-scores), moderate GRM heritability estimates ($h^{2}=0.337\pm 0.079)$ and relevant positional candidate genes.

**Figure 2:**
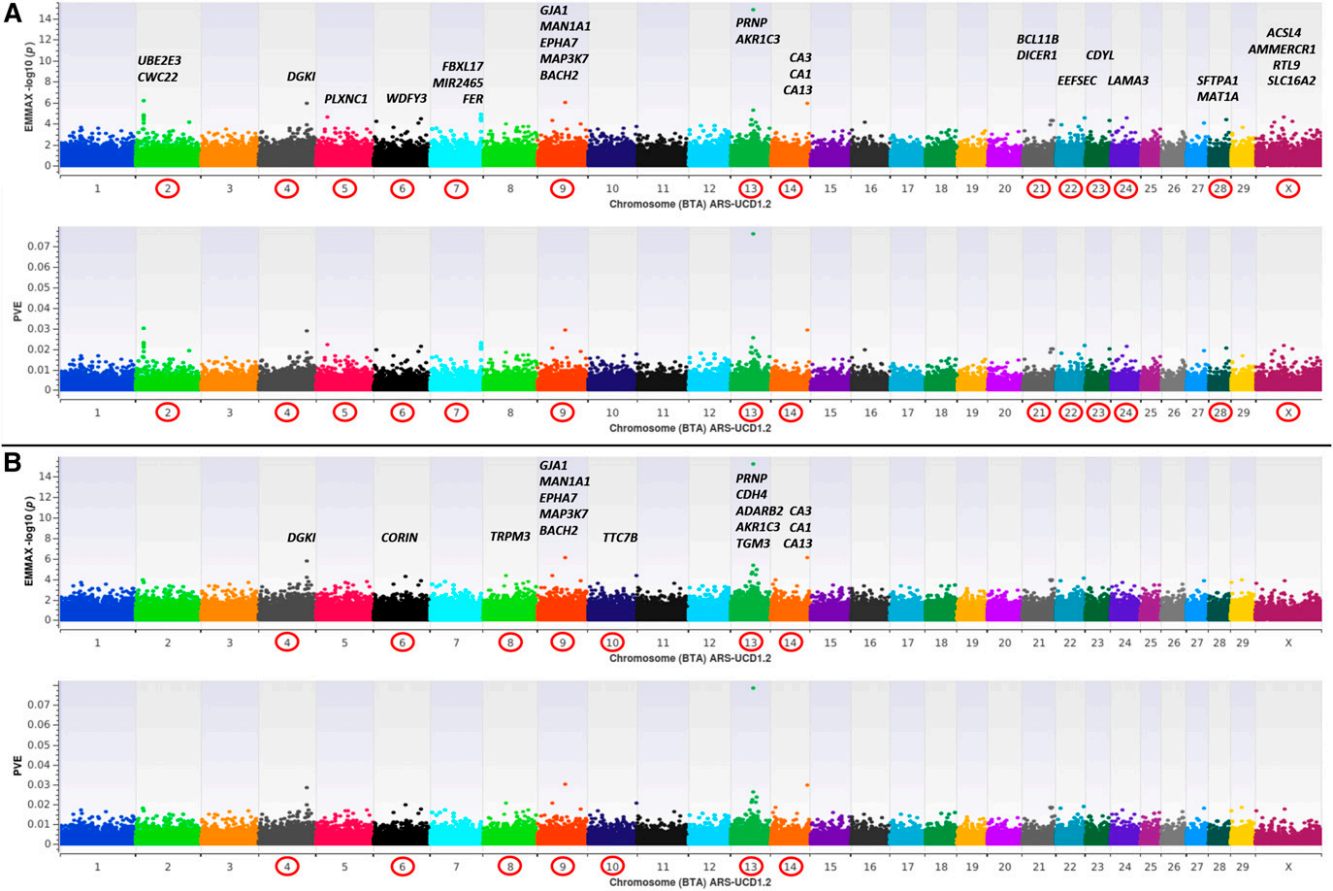
EMMAX genome-wide association analyses (GWAA) for Chronic Wasting Disease (CWD) in farmed U.S. white-tailed deer (*Odocoileus virginianus*; hereafter WTD) using an interval variable (CWD-scores) to simultaneously reflect both the total number of CWD positive diagnostic tissues (*i.e.*, 0, 1, 2) as well as the positive tissue types (*i.e.*, 1 = lymph node only; 2 = lymph node and obex). All dual-panel manhattan plots depict -log10 *P*-values and the proportion of phenotypic variance explained (PVE) by white-tailed deer marker-effects on the y-axis, and the comparative position of all probe sequences on the x-axis, as inferred by blastn alignment with the bovine genome (ARS-UCD1.2). All analyses include diagnostically confirmed CWD positive (n = 284) and CWD non-detect (n = 523) WTD, and marker-based GRM heritability estimates ($\left[h^{2}={\text{σ}}_{g}^{2}/({\text{σ}}_{g}^{2}+{\text{σ}}_{e}^{2})\right]$) (Kang *et al.* 2010; Segura *et al.* 2012; Seabury *et al.* 2017; Smith *et al.* 2019). **a**, EMMAX GWAA for CWD-scores with no fixed-effect covariates, high GRM heritability estimates ($h^{2}=0.589\pm 0.069$) and relevant positional candidate genes. **b**, EMMAX GWAA for CWD-scores with fixed-effect covariates (sex, age, U.S. region), high GRM heritability estimates ($h^{2}=0.515\pm 0.075)$ and relevant positional candidate genes.

Using three reduced representation libraries (n = 374 farmed U.S. WTD) and Illumina paired-end sequencing for reference mapping and variant prediction, we successfully constructed a custom Affymetrix Axiom 200K SNP array for the WTD (See Methods). All probe sequences were aligned to the new PacBio long-read bovine genome assembly (ARS-UCD1.2; GCA_002263795.2), thus creating a comparative marker map. Thereafter, we genotyped a cohort of farmed WTD diagnostically classified (See Methods) as CWD positive (n = 284) and CWD non-detect (n = 523) from three U.S. geographic regions (Midwest, Northeast, South). Genome-wide association analyses were conducted using a mixed linear model with genomic relationship matrix and variance component analysis, thus yielding marker-based heritability estimates (GRM heritability), as implemented in EMMAX (Kang *et al.* 2010; Segura *et al.* 2012). All GWAA’s were carried out using 123,987 quality filtered SNPs for two dependent variables including a binary case-control variable (0 = non-detect; 1 = CWD positive) (Figure 1), and an interval variable (CWD-scores) which simultaneously reflects both the total number of CWD positive diagnostic tissues (*i.e.*, 0, 1, 2) as well as the positive tissue types (*i.e.*, 1 = lymph node only; 2 = lymph node and obex; Figure 2); with non-zero CWD-scores accurately modeling the natural progression of disease (Thomsen *et al.* 2012). Across all GWAA’s (Figure 1, Figure 2), GRM heritability estimates were moderate to high (*i.e.*, $h^{2}=0.337\pm 0.079─0.637\pm 0.070$); thus confirming that differential susceptibility to CWD in WTD is under genetic control, and that host genomic background also influences variation in disease progression. Herein we also confirm the *PRNP* gene as a major risk locus, and specifically, the codon 96 missense variant (G96S; binary case-control *P*-value < 6.30E-11; CWD-scores *P*-value < 1.49E-15) as well as one upstream promoter SNP (Affx-574071595; CWD-scores *P*-value ≤ 5.40E-06) as being significantly associated with differential susceptibility to CWD, and with variation in natural disease progression among WTD (Figure 1, Figure 2, Table S1) (O’Rourke *et al.* 2004; Seabury *et al.* 2007). However, it should also be noted that 11 CWD-positive WTD possessed the codon 96SS genotype, and the proportion of phenotypic variance explained (PVE) by even the largest-effect *PRNP* SNPs detected across all GWAA’s (G96S, Promoter Affx-574071595) was < 0.11 (Figure 1, Figure 2), indicating that loci other than *PRNP* influence differential susceptibility and disease progression. These results are compatible with prior analyses performed in mice; where incubation times for transmissible spongiform encephalopathies were largely influenced by a genetic architecture independent of *PRNP* (Iyegbe *et al.* 2010). Across all GWAA’s (n = 123,987 SNPs), only 61 SNPs met a nominal significance level for polygenic traits (*P*-value ≤ 5E-05) (Wellcome Trust Case Control Consortium 2007; Seabury *et al.* 2017), with 17 detected in more than one GWAA. This is relatively unsurprising since EMMAX is known to produce conservative *P*-values; particularly when large-effect regions exist (Zhou and Stephens 2012). Moreover, the architecture of both investigated CWD traits (Figure 1, Figure 2) is such that few large-effect regions exist (*i.e.*, PVE ≥ 0.03); but together with many smaller-effect regions, a significant proportion of the phenotypic variance can be explained. Interestingly, an investigation of all GWAA’s revealed many of the same positional candidate genes (Figure 1, Figure 2; PVE ≥ 0.02); the majority of which have been implicated in the pathophysiology of various prion diseases including scrapie (*i.e.*, *ACSL4*, *CA3*, *KLF6*), bovine spongiform encephalopathy (*i.e.*, *NPAS3*, *BACH2*, *EPHA7*, *ITGA4*), spontaneous and familial Creutzfeldt-Jakob disease (*i.e.*, *HIST1H4D/OPCML*, *LAMA3*, *TTC7B*), and various other neurodegenerative conditions including Alzheimer’s (*i.e.*, *DGKI*, *SFRP1*, *SLC24A4*) and Parkinson’s disease (*BCL11B*) (Scherzer *et al.* 2007; Tang *et al.* 2009; Tortosa *et al.* 2011; Silver *et al.* 2012; Cohen *et al.* 2013; Lambert *et al.* 2013; Filali *et al.* 2014; Lee *et al.* 2014; Kipkorir *et al.* 2015; Xerxa *et al.* 2016; Esteve *et al.* 2019; Majer *et al.* 2019). Summary data for all GWAA’s and positional candidate genes (Figure 1, Figure 2) as well as the corresponding P-P plots are provided in the supplementary information (Table S1; Additional Files 1-5 in DRYAD).

To investigate the potential for developing a genomically-estimated CWD eradication program for farmed WTD, we used genomic best linear unbiased prediction (GBLUP) in conjunction with *k*-fold cross validation and random sampling (Taylor 2014). Briefly, WTD data (*i.e.*, genotypes, CWD diagnostic phenotypes, and other metadata) were randomly partitioned into *k* -subsamples (*k* = 3, *k* = 5), and one of these subsamples was then selected (*i.e.*, within a discrete fold) to serve as the validation set for genomic prediction; thus the GBLUP model was fit using the remaining (*i.e.*, *k* -1) subsamples within that fold (*i.e.*, the training data); until all subsamples were used for both training and prediction. All cross validations with random sampling were run for 50 iterations, with each iteration consisting of either three or five folds (*k* = 3, *k* = 5), and summary statistics were produced (See Table 1; Methods; Additional Files 6-29 in DRYAD). Binary GBLUP models fit with no fixed-effect covariates (*k* = 3, *k* = 5) produced high mean genomic prediction accuracies (≥ 0.8167) and specificities (≥ 0.9101), with small standard deviations, but lower mean sensitivities (≤ 0.6496), indicating that false negatives pose the greatest challenge for reducing the prevalence of CWD via genomic prediction (Table 1; See Methods). Similar results were also obtained when binary GBLUP models were fit using sex, age, and U. S. region of origin as fixed-effect covariates (*k* = 3, *k* = 5; Table 1). However, in addition to false negatives, some false positive genomic predictions were also observed; most likely due to underlying genomic susceptibility coupled with either very early stages of disease (*i.e.*, CWD non-detect diagnostically) and/or differences in exposure (Tsairidou *et al.* 2014). All results were robust to the inclusion or exclusion of non-autosomal loci (*i.e.*, X, MT; 123,987 SNPs *vs.* 120,808 SNPs, respectively), and to full dosage compensation *vs.* no dosage compensation when putative X-linked SNPs were included (Table 1; Additional Files 6-29 in DRYAD). GBLUP models fit with the CWD-scores (0, 1, 2), thus reflecting the natural progression of disease, produced lower mean genomic prediction accuracies (*i.e.*, ≤ 0.6007; See Methods; DRYAD), regardless of the inclusion or exclusion of fixed-effect covariates (sex, age, U.S. region), non-autosomal loci, or the implementation of full dosage compensation *vs.* no dosage compensation (*k* = 3, *k* = 5).

**Table 1:** Summary of chronic wasting disease genomic predictions in farmed U.S. white-tailed deer (*Odocoileus virginianus*)

*k*-Fold Subsample	GBLUP Model Covariates	Mean AUC (*SD*)*^a^*	Mean Matthews Coefficient (*SD*)	Mean Genomic Prediction Accuracy (*SD*)	Mean Sensitivity (*SD*)	Mean Specificity (*SD*)	Mean RMSE (*SD*)*^b^*
***k* = 3**	None	0.8471 (0.0068)	0.5871 (0.0152)	0.8167 (0.0066)	0.6447 (0.0117)	0.9101 (0.0091)	0.3768 (0.0043)
***k* = 3**	Sex, Age, U.S. Region	0.8534 (0.0063)	0.5787 (0.0158)	0.8119 (0.0070)	0.6735 (0.0122)	0.8870 (0.0081)	0.3746 (0.0040)
***k* = 5**	None	0.8485 (0.0053)	0.5940 (0.0132)	0.8198 (0.0057)	0.6496 (0.0110)	0.9121 (0.0060)	0.3754 (0.0028)
***k* = 5**	Sex, Age, U.S. Region	0.8542 (0.0047)	0.5870 (0.0123)	0.8159 (0.0055)	0.6716 (0.0110)	0.8942 (0.0066)	0.3734 (0.0025)

a Area Under the Curve (AUC; Wilcoxon Mann Whitney Method; See Methods).

b Root Mean Square Error (RMSE; See Methods).

## Conclusions

Herein, we demonstrate that differential susceptibility to CWD and variation in natural disease progression are both heritable, polygenic traits in farmed U.S. WTD, and that genome-wide SNP data can be used to produce accurate genomic predictions for risk (≥ 0.8167); thereby providing the first novel strategy for reducing the prevalence of CWD. Moreover, given the genomic architecture of these traits, we also demonstrate that *PRNP* genotyping alone cannot be expected to facilitate an eradication program, or to rapidly reduce the overall prevalence of CWD in farmed U.S. WTD.

## Author Contributions

C.M.S designed the research with input from T.A.N. M.A.L. and T.A.N. provided CWD diagnostic data, other relevant animal metadata, and biological samples for DNA isolation from existing agency repositories. C.M.S. and D. L. isolated and quantified DNA with assistance from P.F. E.K.B. prepared hair cards and quantified DNA. R.P.M. and C.D.J. prepared and sequenced reduced representation libraries. C.M.S. performed trimming, reference mapping, and variant prediction. C.M.S. designed the Affymetrix Axiom 200K SNP array with assistance from D.L.O. D.L.O. performed blastn searches to create comparative marker maps for the array. D.L.O. submitted reads to NCBI SRA. C.M.S. performed quality filtering of genotypes, genome wide association analyses with marker-based heritability estimates, alignment with positional candidate genes, and genomic predictions with *k*-fold cross validation and summary statistics. C.M.S. wrote the manuscript; incorporating input from D.L.O., E.K.B., D.L., P.F., R.P.M., C.D.J., M.A.L., and T.A.N. All authors edited and approved the manuscript.
